# Risk adjustment in aging societies

**DOI:** 10.1186/s13561-014-0007-5

**Published:** 2014-08-09

**Authors:** Viktor von Wyl, Konstantin Beck

**Affiliations:** CSS-Institute for Empirical Health Economics, Tribschenstrasse 21, Luzern, 6002 Switzerland; Institute for Social and Preventive Medicine, University of Bern, Finkenhubelweg 11, Bern, 3012 Switzerland; Department of Economics, University of Zurich, Blümlisalpstrasse 10, Zurich, 8006 Switzerland

**Keywords:** Risk adjustment, Demography, Health insurance, Intergenerational solidarity, I13, J11

## Abstract

**Background:**

In Switzerland, age is the predominant driver of solidarity transfers in risk adjustment (RA). Concerns have been voiced regarding growing imbalances in cost sharing between young and old insured due to demographic changes (larger fraction of elderly >65 years and rise in average age). Particularly young adults aged 19–25 with limited incomes have to shoulder increasing solidarity burdens. Between 1996 and 2011, monthly *inter* generational solidarity payments for young adults have doubled from CHF 87 to CHF 182, which corresponds to the highest absolute transfer increase of all age groups.

**Results:**

By constructing models for age-specific RA growth and for calculating the lifetime sum of RA transfers we investigated the causes and consequences of demographic changes on RA payments. The models suggest that the main driver for RA increases in the past was below average health care expenditure (HCE) growth in young adults, which was only half as high (average 2% per year) compared with older adults (average 4% per year). Shifts in age group distributions were only accountable for 2% of the CHF 95 rise in RA payments.

Despite rising risk adjustment debts for young insured the balance of lifetime transfers remains positive as long as HCE growth rates are greater than the discount rate used in this model (3%). Moreover, the life-cycle model predicts that the lifetime rate of return on RA payments may even be further increased by demographic changes.

Nevertheless, continued growth of RA contributions may overwhelm vulnerable age groups such as young adults. We therefore propose methods to limit the burden of social health insurance for specific age groups (e.g. young adults in Switzerland) by capping solidarity payments.

**Conclusions:**

Taken together, our mathematical modelling framework helps to gain a better understanding of how demographic changes interact with risk adjustment and how redistribution of funds between age groups can be controlled without inducing further selection incentives. Those methods can help to construct more equitable systems of health financing in light of population aging.

**Electronic supplementary material:**

The online version of this article (doi:10.1186/s13561-014-0007-5) contains supplementary material, which is available to authorized users.

## Background

Societies in highly industrialized countries in Western Europe, North America or Japan have undergone profound demographic changes over the past decades. Life-expectancy has increased substantially, owing to reductions of mortality, better life-styles and greater medical possibilities [1],[2]. It is estimated that the average lifespan in OECD countries rose by more than 6 years between 1970 and 2000 [3]. Although this growing life expectancy is commonly perceived as positive, it puts strains on the welfare systems of industrialized societies. For example, the ratio of retired individuals to active workers is increasingly shifting towards the elderly, and fewer active workers have to support more retired persons [4].

Apart from pension systems, health insurance systems are also affected by those demographic trends. The impact of aging societies – increasing average age and a rising share of elderly (>65 years) – on health care expenditures (HCE) has long been recognized. For example, recent projections of Swiss health care costs adjusted for expected demographic changes predict substantial overall increases in HCE owing to a higher proportion of elderly in the population and higher cost growth for older insured [5]. As for the latter, the higher cost increase for older age groups is well documented by long-term observations. For example, Mendelson and Schwarz analyzed Health Care Financing Administration Data from 1977 through 1987 and noticed a disproportionally high cost growth among the elderly aged 65 and more [6], later termed “steepening” by Buchner & Wasem [7]. Such longitudinal analyses of age-stratified cost-profiles were also performed for Switzerland and reached similar results [8],[9].

However, the reasons for the accelerated cost growth among the elderly are still debated. In particular, the effects of age, medical progress, and interactions thereof are not fully understood, and their disentanglement in statistical models is very challenging. One prominent explanation termed “red herring hypothesis” was put forth by Zweifel et al. [10],[11], which states that the cost increases are not linked to age per se but rather to proximity of death (which is more likely at older age), although opposing studies found significant age effects on HCE increases [12]–[14]. Despite this unresolved debate, there is unanimous agreement that health care expenditures will rise further in the future, which in turn will have implications for health insurance and premium financing.

In settings with competing health insurers and community-rated premiums (e.g. Belgium, The Netherlands, and Switzerland), many of the effects of population aging on health financing are mediated through risk adjustment (RA). Risk adjustment (or risk equalization) is a necessary means to prevent risk selection because individual health care expenditures can vary greatly and in part even predictably, whereas health insurance premiums do not [15]. Therefore, premiums systematically do not match costs for certain age groups (e.g. elderly), which leads to incentives for “cream skimming” and discrimination of insured. Risk selection is also harmful from a societal perspective, because it can create losses in welfare and efficiency [15].

Risk adjustment reduces incentives for risk selection. It operates by estimating the difference between group-specific average health care expenditures and the overall average. The difference between these two amounts is then taxed from groups with below average costs and passed on as a subsidy to groups with greater than average costs. Thus, risk adjustment should equal out risk differences in portfolios of insurers to eliminate “cream skimming”.

In general, risk adjustment leads to re-distribution of money from younger, healthier individuals to older, sicker insured, thereby establishing an *inter* generational solidarity because age is one of the main drivers of risk adjustment transfers (especially in the context of the Swiss risk adjustment formula). Hence, if the share of elderly in a population grows over time (and thus average health care expenditures increase), this means that younger, healthier individuals have to contribute more to risk adjustment in order to achieve risk equalization. Solidarity across age groups is also established by other transfer schemes in mandatory health insurance. In particular, young adults also benefit from tax-financed premium subsidies. But quantitatively risk adjustment is by far the largest solidarity component in mandatory health insurance: In 2012 young adults contributed CHF 1.37 billion to risk adjustment, but received only CHF 0.52 billion in premium subsidies [16]. As a consequence risk adjustment should play a key role in any attempt to re-distribute the burden of rising HCE in aging societies.

Nevertheless, the knowledge on interactions between population aging and risk adjustment is still partial. What is more, frameworks for the implementation of fair (as defined normatively by society) and stable *inter* generational solidarity transfers within risk adjustment are, to our knowledge, still lacking. In this paper, we aim to address two questions. First, we aim to investigate how the Swiss risk adjustment scheme (or any scheme) responds to population aging. We will analyze possible effects both from a cross-sectional (i.e. different age groups at single time-points) and from a lifetime perspective (i.e. following an age-cohort of insured over time). Second, we aim to seek ways how solidarity enforced by risk adjustment can be maintained in long term without financially overwhelming especially vulnerable age groups.

The remainder of this paper is structured as follows. First follows a brief explanation of the Swiss health care setting. Second, to gain a better understanding of the processes leading to an increasing premium burden for young insured we develop a simple model of risk adjustment payments over time from the perspective of young adults, in which we include variables for health care growth and demographic changes. Although the model is generic and can accommodate any age splits we will center these calculations around young adults aged 19 to 25 years for reasons that will be explained in the methods section. The third section outlines the construction of a mathematical model to assess the balance of risk adjustment over a life-cycle. By use of those models from the second and the third section we assess the importance of demographic change for the increase of solidarity transfers from young to old using data from the Swiss risk adjustment statistics. Moreover, we will sketch out ideas on how to reduce and stabilize the levels of risk adjustment payments for specific age groups, again using the young adults as an example. The results section describes applications of the mathematical models within stochastic simulations and tests different reform suggestions for how they reduce the premium burden for 19 to 25 year old insured. The paper concludes with a discussion of the findings.

## Methods

### Setting

The Swiss system of social health insurance is influenced by Enthoven’s concept of managed competition [17]. A large number of health insurers (124 in 1996; 62 in 2011) compete for customers and are obliged to accept any person willing to enroll, independent of age or health status. Mandatory health insurance is organized on a pay-as-you-go basis. Benefit packages are strictly defined and comprehensive. Insurance is not linked to employment, and each insured has to pay the premiums directly to the insurer, although state-funded premium subsidies are granted to individuals in need on the basis of taxable income. Currently 30% of all insured receive such financial assistance [16].

Premiums for mandatory insurance are charged as age-independent community rates with two exceptions. Children between 0 to 18 years of age are granted risk-rated premiums with regard to age on a mandatory basis. Moreover, the health insurance law states that insurance companies can grant premium reductions to young adults aged between 19 to 25 years. An internal risk adjustment scheme (i.e. without supplemental funds from the government), defined by retrospective redistribution of premiums across sex and 15 age groups, was introduced in 1993 and left unchanged until 2011 [18]. Starting in 2012, the Swiss risk adjustment was reformed to be based on prospective payments and to additionally include prior hospitalization as a crude morbidity indicator [19]. Further reform steps are currently discussed in the Swiss parliament and will likely include the introduction of pharmaceutical cost groups.

### Variable notations for modeling analyses

In the following, we will develop a simple model with only two groups of insured, which we term young adults and adults. We are using the following notation:

C = Average health care expenditures

x = Number of individuals

p = Proportion in the general population of insured older than 18 years

a = Risk adjustment payment

Y = Indicator for young adults (e.g. 19–25 year olds)

A = Indicator for adults (e.g. >25 year olds)

i = Indicator variable for the 30 risk groups (ordered by age) that were included in the Swiss risk adjustment scheme until 2011 (15 age groups, male/female). The indicator i = 1,..,k corresponds to risk groups for young adults with the cut-off denoted by k, and the remaining k + 1 to 30 risk groups represent adults.

### Definition of risk adjustment equations

We define average health care expenditures for young adults *Y*, adults *A* and overall as

1 $${\bar{C}}^{Y}=\frac{\sum_{i=1}^{k}C_{i}x_{i}}{\sum_{i=1}^{k}x_{i}}\;{\bar{C}}^{A}=\frac{\sum_{i=k+1}^{30}C_{i}x_{i}}{\sum_{i=k+1}^{30}x_{i}}\;\mathrm{and}\;\bar{C}=\frac{\sum_{i=1}^{30}C_{i}x_{i}}{\sum_{i=1}^{30}x_{i}}$$

The proportion of young adults and of adults is defined, respectively, by

$$p^{Y}=\sum_{i=1}^{k}x_{i}/\sum_{i=1}^{30}x_{i}$$,

and *p*^*A*^ = 1 − *p*^*Y*^. Thus, we can also define average health care expenditures$\bar{C}$as

2 $$\bar{C}=p^{Y}{\bar{C}}^{Y}+\left(1-p^{Y}\right){\bar{C}}^{A}$$

Risk adjustment transfers for young adults and adults can then be written as

3 $$a^{H}={\bar{C}}^{H}-\bar{C}$$

for *H = Y* or *H = A.* We will use equation (3) as starting point for the development of a model of changes in the amount of risk adjustment transfers for young adults.

### Cross-sectional analysis of impact of demographic changes on risk adjustment transfers

For this analysis, we consider the *inter* generational part of risk adjustment transfers defined in equation (3). Given equation (2), we can write the *inter* generational transfer per person for young adults at time point t = 0 as

4 $$a_{0}^{Y}={\bar{C}}^{Y}-\bar{C}={\bar{C}}^{Y}-p^{Y}{\bar{C}}^{Y}-\left(1-p^{Y}\right){\bar{C}}^{A}$$

Note that because${\overset{-}{C}}^{Y}<\overset{-}{C}$risk adjustment transfers for young adults are also negative ($a_{0}^{Y}<0$), meaning that they have to make payments into the fund.

For time points t > 0 the *inter* generational part of risk adjustment transfers becomes

5 $$a_{t}^{Y}={\bar{C}}^{Y}{\left(1+\Delta_{Y}\right)}^{t}-p^{Y}{\left(1+d_{Y}\right)}^{t}{\bar{C}}^{Y}{\left(1+\Delta_{Y}\right)}^{t}-\left(1-p^{Y}{\left(1+d_{Y}\right)}^{t}\right){\bar{C}}^{A}{\left(1+\Delta_{A}\right)}^{t},$$

whereby (1 + Δ_*Y*_)^*t*^ and (1 + Δ_*A*_)^*t*^ stand for average health care expenditure (HCE) growth for young adults and adults with average growth rates of ∆_*Y*_ and ∆_*A*_, respectively. The expression (1 + *d*_*Y*_)^*t*^ denotes average changes in the fraction of young adults in the population of all insured older than 18 years (decrease if d_*Y*_ <0).${\bar{C}}^{Y}$,${\bar{C}}^{A}$and *p*^*Y*^ denote starting point values at time 0 (time index is left away for the sake of simplicity).

We now combine equations (4) and (5) into a difference equation. In addition, we separate the terms into those which are independent of the demographic change *d*_*Y*_ (first row) and those that are dependent on *d*_*Y*_ (second row).

6 $$\begin{array}{l} a_{t}^{Y}-a_{0}^{Y}=[({\bar{C}}^{Y}{\left(1+\Delta_{Y}\right)}^{t}-{\bar{C}}^{A}{\left(1+\Delta_{A}\right)}^{t}]-[(1-p^{Y})({\bar{C}}^{Y}-{\bar{C}}^{A})]\\ \;+p^{Y}[{\left(1+d_{Y}\right)}^{t}({\bar{C}}^{A}{\left(1+\Delta_{A}\right)}^{t}-{\bar{C}}^{Y}{\left(1+\Delta_{Y}\right)}^{t})] \end{array}$$

If *t* = 1, then equation (6) simplifies to

7 $$a_{1}^{Y}-a_{0}^{Y}=\left(1-p^{Y}\right)\left({\bar{C}}^{Y}\Delta_{Y}-{\bar{C}}^{A}\Delta_{A}\right)+d_{Y}p^{Y}\left({\bar{C}}^{A}\left(1+\Delta_{A}\right)-{\bar{C}}^{Y}\left(1+\Delta_{Y}\right)\right)$$

This equation (7) can be transformed into a change rate ∆_*aY*_ through division by$a_{0}^{Y}$.

8 $$\Delta_{\mathrm{aY}}=\left(a_{1}^{Y}-a_{0}^{Y}\right)/a_{0}^{Y}$$

which is important in the context of lifetime redistribution described in the next section. Equation (7) allows for comparative static analyses of the impact of different growth parameter combinations on changes of risk adjustment transfers over time. For example, if there was only a change in the composition of the population (i.e. *d*_*Y*_ ≠ 0 and ∆_*Y*_, ∆_*A*_ = 0), then expression (7) simplifies to$\left(d_{Y}p^{Y}\right)\left({\bar{C}}^{A}-{\bar{C}}^{Y}\right)$. The sign of this simplified expression depends on *d*_*Y*_ only (change in proportion of insured aged 19 to 25 years), which for Switzerland turns out to be negative (<0, c.f. Results). For young adults with negative transfers (*a*^*Y*^ < 0) this means that the amount to be paid increases, because equation (7) becomes even more negative. Alternatively, if the only change present was high cost growth among adults (i.e. ∆_*A*_ > 0 and ∆_*Y*_*, d*_*Y*_ = 0), then expression (7) becomes$\left(p^{Y}-1\right)\left({\bar{C}}^{A}\Delta_{A}\right)$, which is negative because (*p*^*Y*^ − 1) < 0. Finally, high cost growth for adults and an increase in their share of the population (i.e. *d*_*Y*_ <0, ∆_*A*_ > 0 and ∆_*Y*_ = 0) leads to elevated risk adjustment contributions for young adults by the amount of$\left(d_{Y}p^{Y}+p^{Y}-1\right)\left({\bar{C}}^{A}\Delta_{A}\right)+\left(d_{Y}p^{Y}\right)\left({\bar{C}}^{A}-{\bar{C}}^{Y}\right)$, because this expression is negative. A more formal analysis of partial derivatives of equation (7) with respect to different growth parameters is given in Appendix A.1.

To summarize, from our two generation model we can derive the following conclusions with respect to population aging. All other things equal, the risk adjustment debt for young adults increases

if HCE growth rates are higher for adults than for young adults

and/or if the proportion of young adults in the population is shrinking.

### Basic model for lifetime transfers in risk adjustment (cohort analysis)

Given that risk adjustment contributions made by specific age groups can change (as demonstrated in the previous section), what are possible implications for the lifetime balance of risk adjustment transfers? In particular, will the current young generations pay more into risk adjustment than they will ever receive back when they grow old?

In the following we will address those questions by developing a discrete-time overlapping generations model, similar in spirit to those developed for the analysis of pension systems (e.g. [20]). Several features of the Swiss risk adjustment make such an analogy quite fitting. First, Swiss social health insurance, of which risk adjustment is an integral part, is a pay-as-you-go system. Because Switzerland only has an internal risk adjustment system (without any tax-financed contributions as for example in Germany) this means that in any given year the contributions made into the fund must equal the benefits paid (henceforth: “symmetry property”). In addition, risk adjustment transfers follow an age-gradient similar to pension schemes, with younger age groups contributing and older generations profiting from risk adjustment (although the addition of further morbidity-criteria may weaken the age dependency).

**Figure 1:**
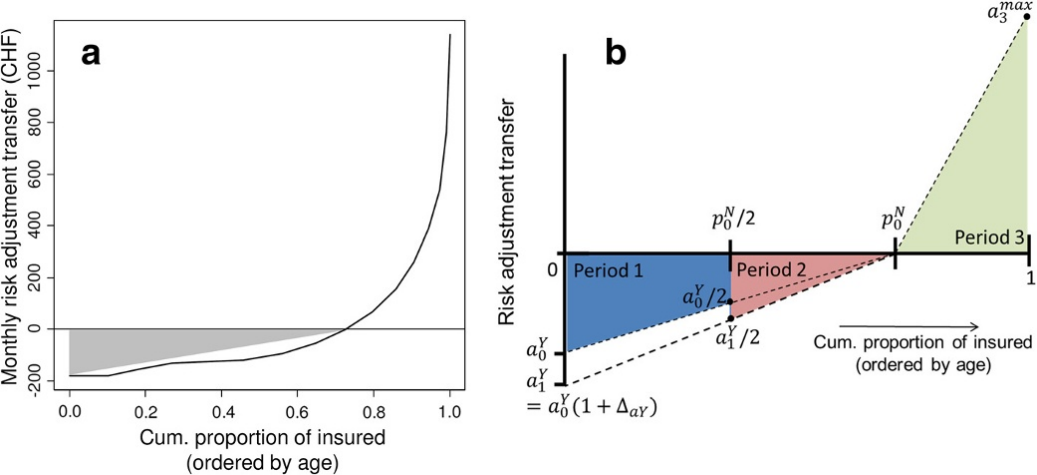
**Schematic drawing outlining the concept for modelling lifetime risk adjustment transfers. a** shows monthly risk adjustment payments in 2011, ordered by amount of payment (which corresponds to increasing age). Because the volume of payments into and out of the fund are symmetric in Switzerland, the area under the curves below and above the zero-line are identical. For the model of lifetime payments the volume of transfers into the fund (and hence out of the fund because of the symmetry) are approximated by a triangle confined by the most negative transfer to the point where the curve of transfers crosses the zero-line on the x-axis. **b** generalizes the model of lifetime payments for 3 time periods (two as a net-payer into and one as a beneficiary of risk adjustment).

The overall idea for the model is as follows. The curve of risk adjustment transfers ordered by amount and weighted by group size roughly resembles two triangles: One below the zero line (net payers aged 19 to 60 years) and one above (net beneficiaries, aged 61 years and older, Figure 1a). As mentioned above, we will make use of the fact that for internal risk adjustment schemes the two areas defined by the zero-line and the risk-adjustment curve are of equal size (symmetry property). Initially, we assume that the shape of those triangles in a given year resembles the pattern of risk adjustment transfers for a single person over a lifetime. In other words, the x-axis interpretation in Figure 1a changes from “age” to “time”. In the simplest model we further assume that a person only lives for two generations: one in which contributions are made and the second in which payments are received. Subsequently we will expand the model to more generations and by modeling growth of risk adjustment payments (Figure 1a and b). The model notation is the same as for the previous section.

#### Two overlapping generations, no population change, undiscounted payments

As a convention the superscripts *N* and *P* denote negative transfers (contributions into fund) and positive transfers (payments from the fund), respectively.

The area of the dotted triangle below the 0 line in Figure 1a ($A_{0}^{N}$at baseline 0) can be approximated by the area of a triangle$(0,p_{0}^{N},a_{0}^{Y}$) in Figure 1b.

9 $$A_{0}^{N}=a_{0}^{Y}p_{0}^{N}/2=A_{0}^{P}$$

The notation is similar as in the previous cross-sectional model, with$a_{0}^{Y}$denoting the risk adjustment of the youngest age group (young adults) and$p_{0}^{N}$representing the proportion of net-payers (aged 19 to 60 years) at baseline (time 0). Because of the symmetry property$A_{0}^{N}=A_{0}^{P}$must hold.

We now turn to the case of two time periods with a cost increase of (1 + *Δ*_*aY*_) between the two periods. The variable ∆_*aY*_ stands for the increase of risk adjustment contributions to be made by young adults in the second time period (cf. Equation 8). Again, there is one time period with payments made into and one with payments received out of the fund. Turning to Figure 1b the young age period with negative transfers is defined by the triangle$\left(0,p_{0}^{N},a_{0}^{Y}\right)$and the old age period by triangle$\left(p_{0}^{N},1,a_{3}^{\max}\right)$. Because of the symmetry property of internal risk adjustment we can define both triangles in terms of$A_{0}^{N}$.

10 $$A_{1}^{N}=A_{1}^{P}=a_{0}^{Y}\left(1+\Delta_{\mathrm{aY}}\right)p_{0}^{N}/2=\left(1+\Delta_{\mathrm{aY}}\right)A_{0}^{N}$$

If Δ_*aY*_ ≥ 0 then it is straightforward to show that the inequality$A_{0}^{N}\leq A_{1}^{N}$holds. This means that the payments made into the fund at time 0 are surpassed by the payments received out of the fund at time point 1.

#### More than two net-payer generations, no population change, present-value perspective

In order to enhance realism of the model we split the life phase of payments to the fund into several separate time periods and allow cost growth between periods. This is shown in Figure 1b for three phases. As a person ages she transits two periods with decreasing contributions into the fund, i.e. the blue area including the points$\left(0,p_{0}^{N}/2,a_{0}^{Y}/2,a_{0}^{Y}\right)$and the red triangle$\left(p_{0}^{N}/2,p_{0}^{N},a_{1}^{Y}/2\right)$, as well as a third life phase with payments out of the fund as shown by the green triangle$\left(p_{0}^{N},1,a_{3}^{\max}\right)$. The term$a_{0}^{Y}/2$follows from the second intercept theorem: If we divide the line (0,$p_{0}^{N}$) of triangle ($0,p_{0}^{N},a_{0}^{Y}$) into halves, then the length of the vertical downward line$\left(0,a_{0}^{Y}\right)$also reduces to$\left(0,a_{0}^{Y}/2\right)$at point$p_{0}^{N}/2$. This implies that the area of triangle ($0,p_{0}^{N}/2,a_{0}^{Y}/2$) is one fourth of the larger triangle ($0,p_{0}^{N},a_{0}^{Y}$), a property used in expression (11).

The steepening dotted black lines symbolize cost growth between different time periods (also note that the area of the red triangle is larger than what the corresponding blue segment for the same period would be). Moreover, we now discount all payments at a rate of *r*.

Let’s first focus on the blue area$\left(0,p_{0}^{N}/2,a_{0}^{Y}/2,a_{0}^{Y}\right)$and the red area$\left(\;p_{0}^{N}/2,p_{0}^{N},a_{1}^{Y}/2\right)$in Figure 1b. We denote the sum of those negative transfers over *n* (here two) time periods by$T_{n}^{N}$.

11 $$T_{n}^{N}=A_{0}^{N}\sum_{i=0}^{n-1}{\left(1+\Delta_{\mathrm{aY}}\right)}^{i}\frac{1}{{\left(1+r\right)}^{i}}\left[{\left(\frac{n-i}{n}\right)}^{2}-{\left(\frac{n-\left(i+1\right)}{n}\right)}^{2}\right]$$

The mechanism of this equation is easy to demonstrate for the case of two periods (*n* = 2) with net contributions into and a third period (*n* + 1) with payments out of the fund. When *i* = 0 then the first term in the square bracket defines the full triangle at time 0$\left(0,p_{0}^{N},a_{0}^{Y}\right)$. In order to obtain the blue segment$\left(0,p_{0}^{N}/2,a_{0}^{Y}/2,a_{0}^{Y}\right)$we have to subtract the smaller triangle$\left(p_{0}^{N}/2,p_{0}^{N},a_{0}^{Y}/2\right)$. The area of the triangle in the second period$\left(p_{0}^{N}/2,p_{0}^{N},a_{0}^{Y}\left(1+\Delta_{\mathrm{aY}}\right)/2\right)$takes cost growth into account. Because of the symmetry property for internal risk adjustment, the area of the green triangle$\left(\;p_{0}^{N},1,a_{3}^{\max}\right)\;$representing insured with positive payments out of the fund at time point 3 (*n + 1*) can be defined according to the following equation.

12 $$T_{n+1}^{P}=A_{n+1}^{P}=A_{0}^{N}{\left(1+\Delta_{\mathrm{aY}}\right)}^{n}\frac{1}{{\left(1+r\right)}^{n}}$$

In order to show that over a lifetime the present value of transfers received from risk adjustment are equal or greater than the amounts paid in we have to verify the following inequality.

13 $$\sum_{i=0}^{n-1}{\left(1+\Delta_{\mathrm{aY}}\right)}^{i}\frac{1}{{\left(1+r\right)}^{i}}\left[{\left(\frac{n-i}{n}\right)}^{2}-{\left(\frac{n-\left(i+1\right)}{n}\right)}^{2}\right]\leq {\left(1+\Delta_{\mathrm{aY}}\right)}^{n}\frac{1}{{\left(1+r\right)}^{n}}$$

It is commonplace in the literature to discount payments at rates between 2% and 3%. In contrast, we estimated the increase of ∆_*aY*_ at 4.5% p.a. between 1996 and 2011 using Swiss risk adjustment statistics ([21], not shown). Under those circumstances the expression${\left(\frac{1+\Delta_{\mathrm{aY}}}{1+r}\right)}^{i}$as a function of *i* is monotonically increasing, which is an important result on the way to proof inequality (13).

In order to demonstrate that expression (13) is true we consider a triangle$\left(0,p_{0}^{N},a_{0}^{Y}\left(1+\Delta_{\mathrm{aY}}\right)\right)$. It is obvious from Figure 1b that the area of this triangle is an overestimate of the actual area of all colored segments, but the mathematical formulation is more tractable. If this triangle is still smaller in size than the one determined by the right hand side of the inequality (green triangle in Figure 1b), then inequality (13) must hold.

The triangle$\left(0,p_{0}^{N},a_{0}^{Y}\left(1+\Delta_{\mathrm{aY}}\right)\right)$corresponds to a situation where the index *i* in (1 + Δ_*aY*_)^*i*^ and$\frac{1}{{\left(1+r\right)}^{i}}$of the left hand side of equation (13) is held fixed at *i* = *n* − 1. This leads to (1 + Δ_*aY*_)^*n* − 1^ and$\frac{1}{{\left(1+r\right)}^{n-1}}$, which are the largest possible values in the iterations (provided that ∆_*aY*_. and *r* are both positive and the function is monotonically increasing). Equation (13) can then be rewritten as follows.

14 $${\left(1+\Delta_{\mathrm{aY}}\right)}^{n-1}\frac{1}{{\left(1+r\right)}^{n-1}}\sum_{i=0}^{n-1}\left[{\left(\frac{n-i}{n}\right)}^{2}-{\left(\frac{n-\left(i+1\right)}{n}\right)}^{2}\right]\leq {\left(1+\Delta_{\mathrm{aY}}\right)}^{n}\frac{1}{{\left(1+r\right)}^{n}}$$

It is easy to show that the sum of the square brackets is just 1 (an expansion of the sum cancels out all terms except for the first and the last, which are 1 and 0, respectively), leading after some re-arrangements to the following equation.

15 $$1\leq \frac{\left(1+\Delta_{\mathrm{aY}}\right)}{\left(1+r\right)}\;$$

Given the observed value for ∆_*aY*_ and an assumed discount rate of 3% inequality (15) holds true. Further note that the right hand side of this inequality can be interpreted as a return rate for the payments made into the fund. Moreover, inequality equation (15) fully integrates with equations (7) and (8) defining ∆_*aY*_, and the discussion of effects of different change parameters on risk adjustment payments for young adults applies. In particular, the expected greater increase in costs for adults (compared with young adults) and the decreasing fraction of young adults in the population (as a result of demographic changes) both increase ∆_*aY*_ and therefore will enhance the return rate.

#### More than two net-payer generations, population change, present-value perspective

It is straightforward to show that the above reasoning can be generalized for any *n* generations of net payers (the return rate will actually remain the same). But what happens if, in addition, the population as a whole gets older in average age? We model this change by introducing an additional growth rate *d*_*N*_, which stands for changes in the proportion of net contributors (aged 19 to 60 years) into risk adjustment.

Turning again to the scenario with multiple time steps on the left hand side and with one time step on the right hand side, we obtain the following inequality.

16 $$\sum_{i=0}^{n-1}{\left(1+\Delta_{\mathrm{aY}}\right)}^{i}{\left(1+d_{N}\right)}^{i}\frac{1}{{\left(1+r\right)}^{i}}\left[{\left(\frac{n-i}{n}\right)}^{2}-{\left(\frac{n-\left(i+1\right)}{n}\right)}^{2}\right]$$

17 $$\leq {\left(1+\Delta_{\mathrm{aY}}\right)}^{n}{\left(1+d_{N}\right)}^{n}\frac{1}{{\left(1+r\right)}^{n}}$$

Again, studying the combination of growth rates${\left(1+\Delta_{\mathrm{aY}}\right)}^{i}{\left(1+d_{N}\right)}^{i}\frac{1}{{\left(1+r\right)}^{i}}$is a key step in the analysis of inequality (16). Because *d*_*N*_ was positive in the past (the fraction of net payers - corresponding to all age groups between 19 and 60 years - increased at 0.33% p.a., although not continuously [21]) it is reasonable to assume that all growth rates of net payer fractions combined as a function of *i* are also monotonically increasing. Proceeding like above we fix all growth rates on the left hand side at *i* = *n* − 1 and at *i* = *n* for the right hand side term of equation (16). After rearrangements and simplifications we obtain inequality (17).

18 $$1\leq \frac{\left(1+\Delta_{\mathrm{aY}}\right)\left(1+d_{N}\right)}{\left(1+r\right)}$$

This inequality (17) holds true if Δ_*aY*_, *d*_*N*_, *r* ≥ 0 and Δ_*aY*_ ≥ *r* or *d*_*N*_ ≥ *r*. Because demographic changes are likely to increase Δ_*aY*_, the return rate on the right-hand side of the equal sign is also expected to become larger, all other things equal.

In summary, the models developed in this section suggest that, all other things equal,

insured will on average receive more (discounted) payments out of the risk adjustment fund than they will have contributed over a lifetime,

and that the expected demographic changes are likely to have an increasing effect on the return rate.

### How can the solidarity burden be redistributed?

The calculations from the basic models suggest that, if unchecked, the solidarity burden for young adults will continue to grow at high rate, which is mainly due to health care expenditure growth among older generations. While those younger generations may still be net beneficiaries of risk adjustment payments over lifetime, their owed risk adjustment debt may nonetheless overwhelm their financial means (cf. [22]). In Switzerland, one such vulnerable group are the young adults aged 19–25 years, which are entitled to premium reductions by law. This relaxation from the community-rate principle was introduced to provide relief for young adults who have limited disposable income. However, by 2011 those rebates have all but disappeared [22]. As already observed by Beck in 2004 [23], the reason for those diminishing rebates is that the Swiss risk adjustment scheme ignores the possibility for premium reductions to young adults and overcharges this age group. This problem has been recognized by federal authorities (e.g. [24]), but no convincing solutions have been presented so far. In particular, many proposed solutions neglect that any reduction of solidarity transfers (be it as a premium reduction or a reduction of risk adjustment contributions) must be compatible with risk adjustment so as not to induce selection incentives for insurers. McGuire et al. [25] and Beck et al. (Beck K, Buchner F, van Kleef R, von Wyl V: *Theory of risk equalization: Are we on the wrong track?* submitted) have provided methodologies for how to limit solidarity transfers within risk adjustment systems. In line with their suggestions we develop a method that can correct for the expected shifts in demography and keep solidarity contributions for specific age groups stable over time. To this end, we introduce two additional parameters in the model described by equation (7).

*γ* = Growth factor determining an upper limit of risk adjustment payment growth. For example, this parameter can be used to decouple risk adjustment contribution growth for young adults from the growth rate of the remaining adults, which is the main driver for the observed increases in younger age groups (c.f. Results).

*ρ* = Factor for reduction of risk adjustment payments (e.g. a premium rebate for young adults). In addition to the stabilization by *γ* a further reduction of nominal payments can be granted. For example, in Switzerland it is currently discussed to charge only 50% of the nominal risk adjustment contributions from 19 to 25 year old individuals (i.e. *ρ* = 0.5) [22].

#### Solution for two risk adjustment groups

In general, the reductions are implemented by subtracting an amount *u*_*t*_ from the nominal RA-payment$a_{t}^{Y}$(defined in Equation 7) so that current RA-payments (at time t) for young adults after correction are equivalent to a baseline payment times a pre-specified growth rate γ.

19 $$a_{0}^{Y}{\left(1+\gamma \right)}^{t}=a_{t}^{Y}-u_{t}$$

Solving for *u*_*t*_ yields equation (19).

20 $$u_{t}=a_{t}^{Y}-a_{0}^{Y}{\left(1+\gamma \right)}^{t}$$

Corrected payments as defined by (18) can be modified further by a second parameter *ρ* that defines the rebate (<1) on nominal RA payments for specific groups (e.g. 50% to 19 to 25 year olds). This reduction is applied to the stabilized risk adjustment contribution$a_{0}^{Y}{\left(1+\gamma \right)}^{t}$for young adults defined in (18), and then equation (19) becomes

21 $$u_{t}^{*}=a_{t}^{Y}-\rho a_{0}^{Y}{\left(1+\gamma \right)}^{t}.$$

Thus, the degree of fairness is determined by the parameters *γ* and *ρ*. The full equation for reduced *inter* generational risk adjustment transfers in young adults reads as

22 $${\tilde{a}}_{t}^{Y}=a_{t}^{Y}-u_{t}^{*}=\rho a_{0}^{Y}{\left(1+\gamma \right)}^{t}$$

For the transfers to sum to zero the payments benefitting older generations must also be shortened by a certain amount. We denote this deduction by *v*_*t*_.

23 $${\tilde{a}}_{t}^{A}=a_{t}^{A}-v_{t}$$

Moreover, we set the restriction that the sum of *inter* generational transfers between young adults and adults must equal to zero according to equation (23)

24 $$\left(1-p_{t}^{Y}\right)\left(a_{t}^{A}-v_{t}\right)+p_{t}^{Y}\left(a_{t}^{Y}-u_{t}^{*}\right)=0$$

Solving for$\left(a_{t}^{A}-v_{t}\right)$and plugging into (22) yields expression (24)

25 $${\tilde{a}}_{t}^{A}=-\frac{p_{t}^{Y}}{1-p_{t}^{Y}}\rho a_{0}^{Y}{\left(1+\gamma \right)}^{t},$$

which corresponds to the new *inter* generational risk adjustment payment of young adults to adults.

An extension of the reasoning in this section to several age groups is given in Appendix A.2. Additionally, in Section A.3 of the appendix we show that the lifetime balance of payments stays positive even after reductions of risk adjustment for specific young age groups.

## Results

### Retrospective analysis of intergenerational solidarity transfers in Switzerland

Next, we illustrate the mathematical models defined by equations (7) and (16) from the methods sections by retrospectively analyzing solidarity transfers between generations over the period of 1996 to 2011 within Swiss risk adjustment. As an example, we center this and the following analysis on the group of young adults aged 19–25 years, and the rationale for that decision is detailed in the methods section and in a companion paper (von Wyl V, Beck K: Distribution of premium burden for mandatory health insurance in Switzerland, submitted).

To inform the models we used data from the official Swiss risk adjustment statistics 1996 through 2011 [21], which are displayed in Table 1. The second column shows the proportion of young adults in the Swiss population of individuals older than 18 years. Over the 15 year observation period this proportion has decreased by 0.8% points, thus the share of young adults has shrunken slightly. Columns 3 and 4 show average costs for the two age groups, whereas columns 5 and 6 represent relative changes using 1996 as the base year (100%). Overall, health care expenditures for young adults have increased by 32% at an average growth rate of 1.88% and even by 78% for adults older than 25 years (average growth rate 3.94%).

**Table 1:** **Evolution of risk adjustment transfers between 1996 and 2011**

		Health care expenditures		Cost increase		Monthly risk adjustment payment into (<0) or from (>0) the fund	
		(CHF per month)		(base year 1996)			
Year	Proportion of young adults	Young adults	Adults	Young adults	Adults	Young adults	Adults
1996	11.1%	61	159	100%	100%	−87	11
1997	10.8%	61	166	101%	105%	−94	11
1998	10.6%	61	175	101%	110%	−101	12
1999	10.6%	62	181	101%	114%	−107	13
2000	10.5%	65	192	106%	121%	−114	13
2001	10.4%	68	203	111%	128%	−121	14
2002	10.5%	69	210	113%	133%	−127	15
2003	10.5%	71	220	117%	139%	−133	16
2004	10.5%	73	234	121%	147%	−143	17
2005	10.5%	75	246	123%	155%	−153	18
2006	10.4%	72	247	118%	156%	−157	18
2007	10.3%	73	257	120%	162%	−165	19
2008	10.3%	76	267	125%	169%	−172	20
2009	10.3%	79	275	129%	173%	−176	20
2010	10.3%	80	280	131%	177%	−180	21
2011	10.3%	80	283	132%	178%	−182	21

Over time, *inter* generational risk adjustment payments for young adults have risen from CHF 87 per month in 1996 to CHF 182 in the year 2011 (Table 1, column 7). Yet the effect of those solidarity transfers on the adults’ side remained rather small. In 1996 each adult received CHF 11 per month in solidarity transfers from 19 to 25 year olds. Fifteen years later those payments have only risen by CHF 10 to a total of CHF 21 per month (Table 1, column 8). Overall the solidarity burden for young adults has experienced the highest absolute growth of any age groups between 1996 and 2011, but with little effect for adults due to the demographic constellation (i.e. much more adults than young adults).

Next, we investigated the importance of the three model parameters (Δ_*Y*_, Δ_*A*_, *d*_*Y*_) for the increase of solidarity payments by young adults. Following the discussion of partial derivatives in Appendix A.1 (equations (A.1.1)-(A.1.3)) we quantified the magnitude of change of RA payments for a one unit parameter increase. To this end we used health care expenditures in 2011 and averages of cost growth and demographic change rates over the 15 year observation period for calculations (Table 1), which yielded values of CHF 21.75, CHF −254.0 and CHF 72.20 for *d*_*y*_*, Δ*_*A*_ and *Δ*_*Y*_, respectively. In other words, solidarity payments owed by young adults are most sensitive to health care expenditure growth among adults, whereas shifts in population composition (i.e. fewer young adults) seem to play a less important role. This finding was confirmed when we performed correlation analyses of observed annual changes in *d*_*y*_*, Δ*_*A*_ and *Δ*_*Y*_ with observed increases in RA contributions of young adults ∆_*aY*_. While spearman correlations of ∆_*aY*_ with *d*_*y*_ (rho = −0.45) and *Δ*_*Y*_ (rho = 0.11) did not reach statistical significance, the HCE growth rate of adults *Δ*_*A*_ showed a strong, statistically significant correlation with ∆_*aY*_ (rho = 0.81, p-value < 0.001).

Along those lines we decomposed the impact of demography on the cumulative observed increase of risk adjustment payments between 1996 and 2011 using equation (7). Over this time period the monthly risk adjustment contribution rose by CHF 95 for young adults (Table 1, column 7). The demography-dependent term of equation (7) sums to CHF 2, whereas the independent terms amount to CHF 93. Thus, 98% of the increase was driven by health care expenditure growth and only 2% were attributable to demographic changes, meaning the decreasing proportion of young adults in the population.

### How will solidarity transfers develop in the future?

Based on the algorithms for RA modifications developed in the methods section and on data presented in Table 1 we performed projections of solidarity transfers between young adults and adults 15 years into the future, starting in 2011 and ending in 2026. Moreover, we tested the impact of two different reform suggestions to relief the premium burden for younger generations. This was done by repeated stochastic simulations based on equation (7). In particular, we used the cost and demography information for the year 2011 from Table 1 as baseline and progressed stepwise in 1 year intervals into the future by randomly selecting, for each analysis year, HCE growth parameters for young adults and adults from the distribution of growth rates observed between 1996 and 2011 (assuming that they came from a Gaussian distribution). The parameter values for the demographic change *d*_*y*_ stemmed from predictions of demographic changes in Switzerland [26] (conservative scenario) and was held fixed at *d*_*y*_ = −0.01234 per year. Subsequently, HCE and risk adjustment contributions were calculated. Each simulation run, consisting of the full 15 year observation period, was repeated 10′000 times.

We explored three scenarios. The first scenario represents the status quo (before the 2012 risk adjustment reform) without any post-hoc modifications of nominal risk adjustment transfers. For the second scenario risk adjustment contributions by young adults were stabilized to increase only with their own HCE growth rate (1 + *Δ*_*Y*_)^*t*^ (note that alternate choices could consider income growth). Thus restriction (18) becomes$a_{0}^{Y}{\left(1+\Delta_{Y}\right)}^{t}=a_{t}^{Y}-u_{t}$.

In the third scenario the risk adjustment payments were further reduced by 50% after capping growth (i.e., *γ = Δ*_*Y*_ and ρ = 0.5), which yields the condition$0.5a_{0}^{Y}{\left(1+\Delta_{Y}\right)}^{t}=0.5\left(a_{t}^{Y}-u_{t}\right)$.

**Figure 2:**
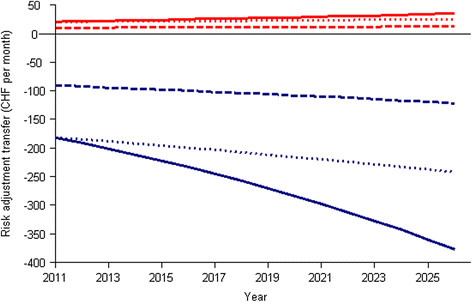
**Projections of risk adjustment transfers from young adults to adults.** Monthly intergenerational risk adjustment transfers of young adults (blue lines) and adults older than 25 years (red lines). The solid lines project transfers without any modifications, and the dashed lines show hypothetical transfers after capping transfer growth for young adults by their health care expenditure growth rate.

The results of those simulations are displayed in Figure 2. The solid lines represent the status quo scenario and suggest that solidarity contributions by young adults (blue line) will grow steadily at a greater-than-linear rate from CHF −182 in 2011 to CHF −378 [95% simulation interval −322; −439] in 2026. In contrast, per-capita contributions received by older generations will increase only moderately from CHF 21 in 2011 to CHF 35 [30; 41] in 2026. The second scenario, which includes a stabilization of RA growth for young adults (dotted line) leads to risk adjustment payments of −244 [−205; −287] by young adults in 2026, whereas adults would receive CHF 25 [21; 30]. If additional reductions on risk adjustment (here: 50%) were granted to young adults this would lead to a solidarity burden of −122 [−103; −143] (starting from CHF −91 in 2011), and adults would receive monthly contributions of CHF 13 [11; 15] (up from CHF 10 in 2011).

Along the same lines we modeled lifetime redistribution through risk adjustment. In particular, we simulated the life cycle of a 19 year old insured starting in 2011 and progressed in one year steps towards the age of 82 (average life expectancy in Switzerland for the year 2011 [27]). Using risk adjustment contributions of the year 2011 as a basis we modeled yearly cost increases for all 15 age groups (by drawing random yearly growth rates from a multivariate normal distribution that was informed with observed cost increases for each age group [21]). All age-specific risk adjustment transfers were summed up and discounted at 3%. In total, 10′000 such life cycles were simulated and repeated for the two different reform suggestions.

Overall, those simulations suggest that for the baseline scenario discounted payments of CHF 83′164 are made into the fund and CHF 114′707 are received out of the fund (difference CHF 30′761 [12′091, 55′467]). When capping risk adjustment payment growth at 2% for young adults the net surplus dropped to CHF 17′069 [2′336, 36′833]. If an additional rebate of 50% on stabilized risk adjustment payments is granted to young adults, then the lifetime balance of discounted payment increased to CHF 19′030 [4′297, 38′795] compared with the scenario without rebate in addition to stabilization.

## Discussion

The view that Western societies will have to face rising healthcare expenditures because of population aging is largely uncontested. Increasing health costs are also observed in Switzerland, which has led to shifts in solidarity transfers over the past years as witnessed by the doubling of risk adjustment payments made by young adults since 1996. In this paper we have analyzed those *inter* generational solidarity transfers between young and old via a mathematical model and estimated the lifetime balance of discounted risk adjustment transfers.

We observed that population aging continuously tends to increase risk adjustment contributions from younger generations. In a model analysis informed by data from Swiss risk adjustment and focusing on young insured aged 19–25 years we found that disparate health care utilization patterns between young and old insured were at the base of the problem of rising solidarity transfers from young adults to older generations. While health care expenditures for young adults grew only moderately, cost growth was almost twice as high among all adults aged 26 years and more. This development has widened the gap of health care expenditure levels between the two age groups. Since risk adjustment operates at closing the gap in order to prevent risk selection, this mechanism has led to increasing *inter* generational solidarity payments owed by young adults.

But how problematic is the rising solidarity burden for younger insured? As for Switzerland, we believe that there is ample societal justification for lowering the burden of risk adjustment payments for young adults aged 19 to 25 years. First and foremost, the current legislation is inconsistent with regards to solidarity contributions by young adults. While the law explicitly allows premium reductions for 19 to 25 year olds, risk adjustment legislation demands that all insured aged 19 and more are treated equal, thus leaving no room for premium rebates.

Second, as outlined in detail in [22], there is mounting evidence that young adults may not be able to carry their solidarity burden, which leads to inefficiencies and inequalities in health financing. Data presented in the companion paper suggest that young adults have to rely heavily on their parents for health insurance premium financing. Along the same lines, the proportion of young adults who are eligible for premium subsidies has risen (although not steadily) from 41% in 2000 (the first year with detailed statistics available) to 44% in 2011 [16].

Third, voting data also presented in the companion paper suggest that the current system with strictly community-rated premiums reflects preferences of net contributors, and young adults in particular, less well than those of older generations who benefit from intergenerational solidarity in the status quo situation (von Wyl V, Beck K: Distribution of premium burden for mandatory health insurance in Switzerland, submitted). It is furthermore remarkable that older generations of today contributed less to solidarity at younger age because of partially age-rated premiums and the possibility for opting-out prior to the reform in 1996.

Finally, it can be argued that the demographic changes and the observed steepening of cost growth across age gradients represent new developments that were not part of voters’ expectations at the time of the referendum for the new health insurance act implemented in 1996, which introduced community rating and a health insurance mandate.

Taken together, those trends have the potential to endanger the generational contract, because they can lead to overwhelming solidarity transfers from young to old (as shown by our model) and thus deteriorate the acceptance of the community-rating principle among the young. Such tendencies are already observed in surveys on the Swiss pension system, which is also under pressure as a result of the demographic change [28].

Consequently, the subject of lowering the growing financial burden of social health insurance for young insured has gained political momentum and has even found its way on the regulator’s agenda. Several reform suggestions have been discussed in the news media, ranging from excluding young adults from risk adjustment, abolishment of premiums for children to relieve young families, or the introduction of new premium age groups for individuals above 50 years to levy more health insurance costs on older generations. What is rarely considered in those suggestions, however, is that in settings with internal risk adjustment (as in Switzerland) such rebates must either be implemented through risk adjustment itself or must at least be reflected, otherwise new selection incentives are created. The premium rebates for young adults are an example of an inconsistent regulation that collided with risk adjustment and led to unwanted results [22].

Within this study we have developed a framework for how such reforms of health premium financing could be implemented in a consistent manner, and we have investigated possible effects on the balance of lifetime risk adjustment payments. We argue that any premium reductions must be implemented directly at the level of risk adjustment payments, thereby giving insurers the possibility to pass on those risk adjustment reductions as premium rebates to young adults. In that regard our method resembles the work of McGuire et al. [25]. Our approach extends theirs by further introducing the concept of stabilization of payments at predefined levels over time. For example, as our analysis has shown risk adjustment payments by young adults increase at a rate that is approximately proportional to health care expenditure growth in older age groups. Without stabilization intergenerational risk adjustment contributions may otherwise soon again rise to levels that exceed disposable means of young adults.

Despite an ever growing solidarity burden, over lifetime the younger generations of today can still expect a net gain from risk adjustment, all other things equal and given that the current level of health care can still be financed in the future. Interestingly, the models suggest that the expected demographic changes may even increase their lifetime rate of return on risk adjustment contributions. As the share of older insured increases over time, former net-recipients of funds from risk adjustment may turn into net-contributors, and therefore the number of net-payers into the fund increases. For example, the group of 51 to 55 year old women still received a small payment from the fund in 1996, whereas 15 years later the same age group has become a net-payer.

In addition, the observed greater average HCE growth for older age groups compared with young adults further increases the return rate of risk adjustment. Similar results are known from modelling studies of pay-as-you-go social security systems [20],[29] or health insurance [30], which found the rate of return also to be dependent on cost growth among older generations (i.e. recipients of payments) and/or increases in the proportion of net payers. Interestingly, an application of generational accounting for health financing to Switzerland corroborates our observation that currently young generations will receive more payments from social health insurance than they will have to contribute over a lifetime [31]. But when these authors performed a more comprehensive analysis including all money streams for health financing the lifetime balance of payments turned negative for several younger age groups.

Although developed for the Swiss setting, our models of demographic effects on risk adjustment and on the life-time balance of transfers also apply to other countries with risk adjustment, such as the Netherlands or Germany. But since both countries raise premiums for mandatory insurance in an income-dependent manner, the direct effects of demographic changes on risk adjustment will be less felt than in Switzerland (whereas health financing of those countries will be more affected by the expected decrease of the ratio of active to retired workers).

Similar to our framework for controlling age-dependent solidarity transfers, the U.S. has implemented a rule that age-based premiums are only allowed to vary within a ratio of 3 (oldest age group) to 1 (youngest) [25]. In contrast, solidarity restrictions between age groups as a means to improve equity are usually not needed in settings with income-dependent premiums.

However, our modelling findings hinge on assumptions regarding rates of risk adjustment growth, the proportion of net payers, and discount rates. Also, further modifications to risk adjustment such as the planned introduction of pharmaceutical cost groups in Switzerland will have a yet unclear impact on the balance. For example, the proportion of net payers *p*^*N*^ may suddenly change if additional morbidity criteria are introduced into the risk adjustment model. Moreover, the impact of population aging on the parameters ∆_*aY*_ and *p*^*N*^ is not continuous and difficult to predict in the long run. An aging population drives up the population average of HCE from which risk adjustment contributions are measured, and age groups which have formerly received payments from risk adjustment may become net-contributors in subsequent years. Finally, our model focused only on risk adjustment payments and ignored any other components of health financing such as direct and indirect taxes, private insurance, and premium subsidies. Furthermore, it should be noted that our model does not address issues of sustainability and affordability of health finance. Generational accounting may offer suitable tools for such more comprehensive analyses [32].

## Conclusions

In summary, the framework developed in this paper may help to construct a more equitable system of health financing in light of population aging and to strengthen the acceptance of the intergenerational contract in social health insurance systems with community-rating.

## Appendix

### A.1 Analysis of change rates

We now analyze equation (7) by calculating the partial derivatives of$\left(a_{1}^{Y}-a_{0}^{Y}\right)$with respect to the variables *d*_*Y*_, *Δ*_*Y*_, *Δ*_*A*_.

26 $$\partial \left(a_{1}^{Y}-a_{0}^{Y}\right)/\partial d_{Y}=p^{Y}\left({\bar{C}}^{A}\left(1+\Delta_{A}\right)-{\bar{C}}^{Y}\left(1+\Delta_{Y}\right)\right)$$

This partial derivative (A.1.1) indicates that changes in risk adjustment contributions resulting from demographic trends are also dependent on the absolute difference of cost levels between young adults and adults at time t=1. Because HCE and growth rates of adults are larger than those of young adults, i.e.${\bar{C}}^{A}\left(1+\Delta_{A}\right)>{\bar{C}}^{Y}\left(1+\Delta_{Y}\right)$expression (A.1.1) is positive. Going back to equation (7) describing the change in risk adjustment payments for young adults it becomes apparent that an increase in the proportion of young adults by *d*_*Y*_ reduces their risk adjustment debt, because this will make expression (7) less negative.The partial derivative with respect to HCE growth for adults is as follows.

27 $$\partial \left(a_{1}^{Y}-a_{0}^{Y}\right)/\partial \Delta_{A}=\left(d_{Y}p^{Y}+p^{Y}-1\right){\bar{C}}^{A}$$

As long as *p*^*Y*^ represents a minority this expression is negative since (*d*_*Y*_*p*^*Y*^ + *p*^*Y*^ − 1) < (2*p*^*Y*^ − 1) < 0, which means that all other things equal a one unit increase in HCE growth for adults leads to an increase of the risk adjustment debt for young adults.

Equation (A.1.3) shows the partial derivative with respect to HCE growth in young adults.

28 $$\partial \left(a_{1}^{Y}-a_{0}^{Y}\right)/\partial \Delta_{Y}=-\left(d_{Y}p^{Y}+p^{Y}-1\right){\bar{C}}^{Y}$$

Expression (A.1.3) has the inverse sign of (A.1.2). Thus, all other things equal, a one unit increase in HCE growth for young adults decreases their risk adjustment debt.

### A.2 Solidarity reductions for several risk adjustment groups

The algorithm described in equations (18)-(24) can also accommodate reductions for several age groups *i* = 1,..,m. For the simple case where modifications of RA transfers only apply to net contributors to risk adjustment this can be written as

29 $${\tilde{a}}_{i,t}=a_{i,t}-u_{i,t}^{*}$$

for age groups *i* = 1,..,m.

The solidarity cap is implemented according to equation (21), whereby *ρ*_*i*_ is set to 1 if no post-hoc modifications to risk adjustment payments are applied. Likewise, if no modifications to *γ*_*i*_ apply then it can simply be set to 0.

30 $$u_{i,t}^{*}=a_{i,t}-\rho_{i}a_{i,0}{\left(1+\gamma_{i}\right)}^{t}$$

Next, we derive the solidarity transfers for older age groups m+1 to 30. We denote those groups of beneficiaries from intergenerational solidarity transfers by subscript *r* and treat them as one group (for sake of simplicity). As in the simple case with only two age groups discussed above the sum of *inter* generational solidarity transfers must equal to 0 in internal risk adjustment systems, and therefore the new transfers from young insured to the (old) reference group can be written as

31 $${\tilde{a}}_{r,t}=-\sum_{i=1}^{m}\frac{x_{i,t}}{x_{r,t}}\rho_{i}a_{i,0}{\left(1+\gamma_{i}\right)}^{t},$$

with *r* symbolizing the reference population consisting of all m+1,…,30 remaining groups and the variables *x*_*i,t*_ and *x*_*r,t*_ being the number of individuals in group *i* and the reference group *r*, respectively.

Modifications of transfers within recipients of RA payments (e.g. higher payments for specific age groups) are also possible, but they require an additional calculation step, in which the total volume of intergenerational RA-payments from younger age groups are redistributed within older generations. This can be achieved, for each age group j = m+1 to 30, by defining weights *w*_*j,t*_ according to equation

32 $$w_{j,t}=\frac{x_{j,t}}{x_{r,t}}\frac{\pi_{j}}{\sum_{j=m+1}^{30}\pi_{j}},$$

whereby *π*_*j*_ denotes the group specific factor (analogous to *ρ*_*i*_).

### A.3. Implication of restricted solidarity for lifetime redistribution

How is the lifetime balance of payments into and from the risk adjustment fund affected by the modifications proposed in equations (18) to (24)? As defined by equation (18) rebates are defined as a reduction of nominal payments. By use of inequality equation (16) the lifetime balance in settings with premium reductions for specific age groups *g* (which also correspond to specific time periods as an individual transits through all age groups over lifetime) can therefore be defined by

33 $$T_{n}^{N}-a_{0}^{Y}\sum_{i\in g}{\left(1+\Delta_{\mathrm{aY}}\right)}^{i}{\left(1+d_{N}\right)}^{i}\frac{1}{{\left(1+r\right)}^{i}}\left[{\left(\frac{n-i}{n}\right)}^{2}-{\left(\frac{n-\left(i+1\right)}{n}\right)}^{2}\right]\rho_{i}$$

34 $$\leq T_{n+1}^{P}-a_{0}^{Y}{\left(1+\Delta_{\mathrm{aY}}\right)}^{n+1}{\left(1+d_{Y}\right)}^{n+1}\frac{1}{{\left(1+r\right)}^{n+1}}\sum_{j\in g}\left[{\left(\frac{n-j}{n}\right)}^{2}-{\left(\frac{n-\left(j+1\right)}{n}\right)}^{2}\right]\rho_{j}$$

Note that the indices *i* and *j* have somewhat different interpretations. The left-hand side of equation (A.3.1) sums up all payments made over *n* time periods, and hence *i* refers to time. The right hand side of equation (A.3.1) represents, according to our convention, the volume of all contributions made by net payers at time *n+1*. Hence the parameter *j* has the interpretation of age groups. This distinction does not alter the conclusions, however.

We have already demonstrated that the inequality$T_{n}^{N}\leq T_{n+1}^{P}\;$holds if the combination of growth rates yields a monotonically increasing function. Therefore we only have to proof that the reductions up to period *n* are smaller than the sum of all reductions at time point *n+1*. We will first demonstrate this for constant, time-independent reductions *ρ*. Since all factors before the sum sign combined are monotonically increasing we can fix *i* at *n* for all growth rates on the left-hand side of equation (A.3.2), which makes them constant.

35 $$a_{0}^{Y}{\left(1+\Delta_{\mathrm{aY}}\right)}^{n}{\left(1+d_{N}\right)}^{n}\frac{1}{{\left(1+r\right)}^{n}}\sum_{i\in g}\left[{\left(\frac{n-i}{n}\right)}^{2}-{\left(\frac{n-\left(i+1\right)}{n}\right)}^{2}\right]\rho_{i}$$

36 $$\leq a_{0}^{Y}{\left(1+\Delta_{\mathrm{aY}}\right)}^{n+1}{\left(1+d_{Y}\right)}^{n+1}\frac{1}{{\left(1+r\right)}^{n+1}}\sum_{j\in g}\left[{\left(\frac{n-j}{n}\right)}^{2}-{\left(\frac{n-\left(j+1\right)}{n}\right)}^{2}\right]\rho_{j}$$

Because all elements of the sums over *i* and *j* are time-independent and the same on both sides of the equation they can be dropped. Thus, the remaining inequality must hold true given our assumption of monotonically increasing growth terms in combination.

Using the same approach, it is simple to demonstrate that the inequality also holds when growth of$a_{0}^{Y}$is capped by a pre-defined, positive growth rate *γ*_*i*_ (by replacing the parameter ∆_*aY*_ in equation (A.3.2), proof not shown).

## Authors’ original submitted files for images

Below are the links to the authors’ original submitted files for images. Authors’ original file for figure 1 Authors’ original file for figure 2
