# Supplementary figures and images for: Risk adjustment in aging societies
Source: Health Econ Rev. 2014 Aug 9;4:7. doi: 10.1186/s13561-014-0007-5 (PMC4884009; doi:10.1186/s13561-014-0007-5)

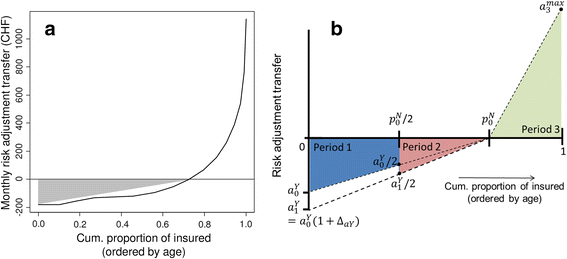

Supplement: Supplementary file 1 — Authors’ original file for figure 1 [file 13561_2014_7_MOESM1_ESM.gif]

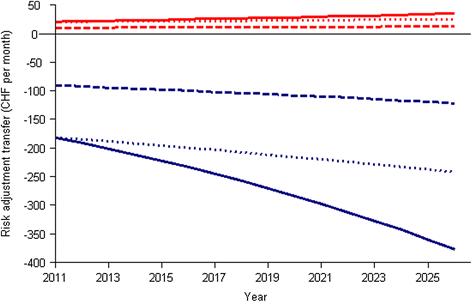

Supplement: Supplementary file 2 — Authors’ original file for figure 2 [file 13561_2014_7_MOESM2_ESM.gif]
